# Plasma complement C3 and C3a are increased in major depressive disorder independent of childhood trauma

**DOI:** 10.1186/s12888-022-04410-3

**Published:** 2022-11-29

**Authors:** Xianmei Luo, Zeman Fang, Lingyun Lin, Haiyun Xu, Qingjun Huang, Handi Zhang

**Affiliations:** 1 Mental Health Center of Shantou University, Mental Health Center, Taishan North Road, Shantou, 515065 China; 2 The Fourth People’s Hospital of Chengdu, Chengdu, China; 3 Affiliated Kangning Hospital, School of Psychiatry, Wenzhou Medical University, Wenzhou, China

**Keywords:** C3, C3a, C1q, CRP, Major depressive disorder, Childhood trauma

## Abstract

**Background:**

Dysregulated complement system is linked to pathophysiology of major depressive disorder (MDD). Childhood trauma has been associated with an increased incidence of adult depression via a putative mechanism of immune activation. This study aimed to measure and compare peripheral levels of complement C3, C3a, C1q and C-reactive protein (CRP) in MDD patients and healthy controls and explore the relationship between these molecule levels and childhood trauma history in the participants.

**Methods:**

The participants were 49 medication-free MDD patients and 45 healthy controls. All participants were asked to finish the Childhood Trauma Questionnaire, followed by blood sampling for measurement of plasma complement C3, C3a, C1q and CRP by means of enzyme-linked immunosorbent assay.

**Results:**

Peripheral plasma concentration of C3 and C3a in medication-free MDD group was significantly higher than that in the healthy controls; whereas the concentration of plasma C1q and CRP in depressed patients was comparable to that in healthy controls. All these inflammatory factors were not associated to childhood trauma experience in patients with MDD.

**Conclusion:**

Our data suggest that complement C3 and C3a may be implicated in the pathophysiology of MDD, although traumatic childhood experiences were not associated with the circulating levels of complement C3, C3a, C1q and CRP.

**Supplementary Information:**

The online version contains supplementary material available at 10.1186/s12888-022-04410-3.

## Background

Major depressive disorder (MDD) is a common mental illness, with almost one in five people experiencing one depressive episode at some point in their lifetime [1]. The disease severely limits patients’ psychosocial functioning and diminishes their quality of life [2]. However, its pathophysiological mechanisms remain poorly understood. In recent years, accumulating evidence supports the involvement of inflammatory factors in the pathophysiology of depression. For example, increased levels of C-reactive protein (CRP), interleukine-6 (IL-6) and tumor necrosis factor-α have been repeatedly observed in patients with depression [3, 4]. Moreover, a recent meta-analysis indicates that anti-inflammatory agents show a significant antidepressant effect either as add-on treatment or as monotherapy for depressive patients [5].

The complement components are essential immune regulators participating in the native inflammatory immune defensive process majorly occurred in the peripheral system [6]. Recent studies consistently demonstrate that complement system also plays an important role in the development and plasticity of the central nervous system [7, 8]. During the development of the retinogeniculate system, excess synapses are removed via complement-mediated synaptic elimination [8]. Furthermore, the aberration of complement activation may be one of the key pathophysiological mechanisms for some neuropsychiatric diseases [9–11]. It has been demonstrated that the inappropriately activated component system mediates the synaptic loss in mouse models of Alzheimer’s disease [9]. In addition, complement proteins are involved in microglia-mediated synaptic pruning in a mouse model of frontotemporal dementia [11]. Synaptic loss and deficits in functional connectivity is associated with MDD [12]. However, the role of complement system molecules in the pathophysiology of depression is not fully clarified.

Complement system consists of over 30 proteins that are activated in a cascade manner through three major pathways: classical, alternative, and mannose-binding-lection. All pathways converge on the cleavage of the complement component C3. C3 is cleaved into two active products, C3a and C3b, which further activate downstream cascade components and participate in immunomodulatory process [13]. Although elevated C3 levels were found in patients with depression [14–17], inconsistent results were also reported [18–22]. C3a is the major active product of C3 and recently found to be implicated in the impairment of blood–brain barrier integrity [23], which is considered as a potential pathophysiology of MDD [24]. Furthermore. C3a receptor signaling mediates chronic stress-induced depressive-like behaviors in an animal model of depression [25]. Although these accumulating evidences suggest that C3a may play an important role in the pathogenesis of MDD, to our knowledge, the levels of C3a in MDD patients have not been investigated. In addition, complement C1q is the initiating protein in the classical complement cascade. Previous studies demonstrate that C1q plays a key role in CNS synapse elimination and neuroprotection [8]. Mice deficient in C1q or the downstream C3 exhibit sustained defects in synapse elimination [8]. However, the relationship between C1q and MDD has been rarely investigated.

It is well known that childhood trauma has long lasting adverse effects on mental health [26, 27] and is closely linked to the increased risk of MDD [28]. Immunoinflammatory activation has been recognized as a potential biological mechanism mediating the devastating effects of childhood trauma on mental health [29]. Recent meta-analyses and systemic reviews have provided strong evidence that peripheral inflammatory factors, such as proinflammation cytokines and acute phase proteins, are significantly associated with adverse childhood experience [30, 31]. However, to our knowledge, the relationship between childhood traumas and the expression and function of complement system molecules and their possible impact on the clinical characteristics of MDD have not been explored so far.

The aim of this study was to investigate if plasma concentrations of C3, C3a, C1q and CRP in patients with MDD differ from those in healthy controls. Furthermore, we performed an exploratory analysis on the possible correlations of complement components and CRP with the experience of childhood trauma and other demographic and clinical variables.

## Methods

### Participants

A total of 54 MDD patients admitted to the Mental Health Center of Shantou University were enrolled for this study. The diagnosis was established according to the 10^th^ version of International Classification of Diseases (ICD-10). Patients did not take any medication for at least two weeks before the onset of this study. Patients were excluded if they were (1) < 16 or > 65 years old, (2) suffered other central nervous system diseases, autoimmune disorders, endocrine diseases, infectious diseases, or other severe physical diseases, (3) alcohol or drug dependent, (4) comorbidity with other psychiatric disorders except for anxiety disorder. Forty-five healthy adults were recruited as healthy controls (HC) in the study. Most of them were employees in the hospital and college students. The same exclusion criteria were applied to HC and patients. The study protocol was approved by the Human Ethics Committee of the Mental Health Center of Shantou University (the approval number: 201906). All subjects signed an informed written consent.

### Clinical assessment

The demographic information and clinical characteristics were collected from all patients and HC. Severity of depression and anxiety was assessed using the Hamilton Rating Scale for Depression (HAMD)-24 [32] and the Hamilton Rating Scale for Anxiety (HAMA) [33], respectively. The presence of childhood trauma was retrospectively assessed using the Childhood Trauma Questionnaire (CTQ) with 28 items [34], which includes five subscales: Emotional Abuse, Physical Abuse, Sexual Abuse, Emotional Neglect, and Physical Neglect. Each subscale is composed of 5 items, and each item rates on a 5-point scale regarding frequency of occurrence of traumatic events (from ‘1 = never true’ to ‘5 = very often true’). The remaining three items are set as a weighted score of CTQ. The total score of each subscale ranges from 5 to 25. The total score of CTQ is from 25 to 125, which is associated with severity of child trauma. We used the Chinese version of the CTQ [35], which has been widely used in previous studies and showed a good reliability and validity [36, 37].

### Blood tests

Four ml of venous blood was drawn using tubes containing ethylene diamine tetraacetic acid as anticoagulant. Then blood sample was centrifuged at 1500 rpm for 10 min at 4℃. All plasma samples were then stored at -80℃ until test. Complement C3 and C1q were measured by Human Complement C3 enzyme linked immunosorbent assay (ELISA) Kit (Cat No. ab108823; Abcam, Cambridge, UK) and Human Complement C1q ELISA Kit (Cat No. ab170246; Abcam, Cambridge, UK), respectively. C3a was measured with Human C3a ELISA kit (Cat No. BMS2089; Thermo Fisher Scientific, MA, USA), and CRP was measured with Human C-Reactive Protein/CRP Immunoassay Quantikine® ELISA (Cat No. DCRP00; R&D Systems, Inc. Minneapolis, USA). Each plasma sample was measured in duplicate following the manufacturer’s protocols.

### Statistical analysis

Data of continuous variables that conform to a normal distribution are reported as mean ± standard deviation, and data that do not conform to the normal distribution are represented as median (interquartile range). The categorical variable is expressed as a percentage. For group comparisons, categorical data were analyzed using chi-squared (χ^2^) test. Non-parametric data were analyzed by Mann–Whitney U tests. When comparing complement levels between the MDD and HC groups, general linear model analysis was used to control for age and body mass index (BMI). The spearman association analysis was used to examine the correlation of peripheral levels of complement components and demographic variables. Multiple linear regression was used for correlational analysis of complementary components and CRP with childhood trauma experiences and clinical variables with controlling demographic variables and diagnosis. Results were considered statistically significant at *P* < 0.05. Statistical analysis was performed with SPSS 21.0.

## Results

### Participant characteristics

Five cases with the plasma concentrations of CRP greater than 10,000 ng/ml were excluded since CRP over 10,000 ng/ml indicates a sign of infection. All excluded cases are in MDD group and no case in HC group is excluded. Thus, a total of 49 patients with MDD and 45 HC were included in the analysis. Demographic and clinical data for MDD and HC groups are illustrated in Table 1. There were no statistically significant differences between the two groups in gender distribution and marriage status. Patients were marginally younger and their BMI was lower than the controls, and the differences are statistically significant. In terms of clinical characteristics, patients reported an average onset of MDD at 21.0 (10.0) years old and the illness lasted for 1.5 (2.1) years. The mean scores of HAMD and HAMA were 30.2 ± 6.0 and 22.4 ± 7.9, respectively.

**Table 1:** Sample characteristics

	MDD (*n* = 49)	HC (*n* = 45)	Statistic	*P* value
Age (year)	22 (13)	25 (5)	U = 1395.0	**0.027**
Female gender	75.5%	73.3%	χ^2^ = 0.058	0.809
Married	22.4%	22.2%	χ^2^ = 0.001	0.979
BMI (kg/m^2^)	19.22 (5.26)	20.70 (2.96)	U = 1415.5	**0.018**
Age of onset (year)	21.0 (10.0)	-	-	-
Duration of illness (year)	1.5 (2.1)	-	-	-
HAMD-24	30.2 ± 6.0	-	-	-
HAMA	22.4 ± 7.9	-	-	-

*MDD* Major depressive disorder, *HC* Healthy controls, *BMI* Body mass index, *HAMD-24* The hamilton rating scale for depression-24, *HAMA* The hamilton rating scale for anxiety

As shown in Table 2, the MDD group had a significantly higher total CTQ score than HC. The scores in the four subscales, including Emotional Abuse, Emotional Neglect, Physical abuse, and Physical neglect, were also higher in the MDD group compared to the HC group. But there was no significant difference in sexual abuse between the two groups.

**Table 2:** The scores of CTQ and sub-CTQ scales

	MDD (*n* = 49)	HC (*n* = 45)	U	*P*
CTQ total score	48 (22)	38 (6)	294.0	** < 0.001**
Emotional Abuse	10 (7)	7 (3)	446.5	** < 0.001**
Emotional Neglect	13 (7)	7 (3)	288.5	** < 0.001**
Physical Abuse	8 (5)	5 (2)	524.5	** < 0.001**
Physical Neglect	11 (6)	6 (2)	332.0	** < 0.001**
Sexual Abuse	5 (1)	5 (0)	1023.0	0.432

*MDD* Major depressive disorder, *HC* Healthy controls, *CTQ* Childhood trauma questionnaire

### The plasma levels of C3, C3a, C1q and CRP

C3 and C3a levels in depressed patients were significant higher compared to HC, while levels of C1q was comparable between patients and controls. CRP levels was lower in depressed patients than that in HC (Table 3). After controlling for age and BMI, the two groups were still significantly different in C3 (F = 8.021, *P* = 0.006) and C3a levels (F = 14.449, *P* < 0.001), but not in levels of CRP (F = 2.087, *P* = 0.152) and C1q (F = 0.322, *P* = 0.572).

**Table 3:** Levels of plasma complements and CRP

	MDD (*n* = 49)	HC (*n* = 45)	U	*P*
C3 (mg/ml)	6.68 (5.22)	4.96 (4.28)	830.0	**0.039**
C3a (ng/ml)	572.21 (479.22)	467.27 (208.58)	697.5	**0.002**
C1q (μg/ml)	180.98 (78.73)	213.95 (137.59)	1282.0	0.174
CRP (ng/ml)	114.68 (454.67)	442.35 (942.07)	1457.5	**0.007**

*C3* Complement component 3, *C3a* Complement component 3a, *C1q* Complement component 1q, *CRP* C-reactive protein

### Correlation of peripheral complement components and CRP with CTQ, demographic and clinical variables

Spearman correlation analysis showed increased levels of C3, C3a and CRP were significantly associated with higher BMI in MDD patients, while only CRP was correlated with BMI in the HC group (Table 4). In addition, C3 levels were positively correlated with age in the HC group (Table 4). Levels of C3, C3a and CRP, but not C1q were significantly associated with BMI, and all these inflammatory factors were not associated with age in all participants with controlling for diagnosis (Table 4). Contrary to our prediction, the concentration of C3, C3a, C1q and CRP were not correlated with the total CTQ score or any CTQ subscale scores in the MDD group after controlling for age and BMI (Table 5). In the HC group, C1q levels were correlated with emotional abuse and C3 levels were associated with emotional abuse, emotional neglect, physical neglect and CTQ total score after controlling for age and BMI (Table 5). No correlations between complementary components and CRP with childhood trauma experiences were found in all participant with controlling for diagnosis, BMI, and age (Table 5).

**Table 4:** Associations of plasma complements and CRP with age and BMI in the MDD group, HC group, and all participants

		C3			C3a			C1q			CRP		
		MDD*	HC*	ALL^#^	MDD*	HC*	ALL^#^	MDD*	HC*	ALL^#^	MDD*	HC*	ALL^#^
Age	r_s_/β	-0.026	0.306	0.117	0.042	0.099	-0.086	0.132	0.103	0.070	0.170	-0.085	0.054
	P	0.861	**0.041**	0.254	0.775	0.519	0.390	0.364	0.501	0.513	0.244	0.579	0.566
BMI	r_s_/β	0.405	0.170	0.261	0.385	0.055	0.233	0.146	0.216	0.182	0.533	0.417	0.431
	P	**0.004**	0.265	**0.015**	**0.006**	0.718	**0.026**	0.318	0.154	0.101	** < 0.001**	**0.004**	** < 0.001**

^*^ Spearman correlational analysis was used for the MDD and HC group, and spearman r (rs) is present in the table

^#^ Multiple linear regression (MLR) was used for correlational analysis in all participants to control for the diagnosis, and standardized beta (β) is present in the table; plasma complements and CRP were LN-transformed in MLR. *C3* Complement component 3, *C3a* Complement component 3a, *C1q* Complement component 1q, *CRP* C-reactive protein, *MDD* Major depressive disorder, *HC* Health control, *ALL* All participants

**Table 5:** Associations of plasma complements and CRP with childhood trauma experiences in the MDD group, HC group, and all participants

		C3*			C3a*			C1q*			CRP*		
		MDD	HC	ALL	MDD	HC	ALL	MDD	HC	ALL	MDD	HC	ALL
CTQ total score	β	-0.040	-0.339	-0.176	0.121	-0.191	0.056	0.074	-0.232	-0.061	-0.001	-0.112	-0.046
	P	0.773	**0.019**	0..146	0.391	0.220	0.641	0.611	0.130	0.634	0.995	0.441	0.683
Emotional Abuse	β	-0.010	-0.310	-0.102	0.142	-0.230	0.084	0.026	-0.342	-0.123	0.041	-0.052	0.008
	P	0.946	**0.033**	0.377	0.320	0.138	0.458	0.862	**0.023**	0.309	0.755	0.724	0.941
Emotional Neglect	β	-0.082	-0.350	-0.216	0.167	-0.130	0.116	0.051	-0.078	-0.002	-0.050	-0.113	-0.077
	P	0.567	**0.015**	0.087	0.244	0.406	0.351	0.736	0.613	0.989	0.704	0.441	0.516
Physical Abuse	β	0.017	-0.245	-0.092	0.105	-0.169	0.036	0.046	-0.226	-0.081	-0.066	0.017	-0.040
	P	0.902	0.096	0.395	0.456	0.277	0.738	0.755	0.141	0.475	0.610	0.986	0.689
Physical Neglect	β	-0.028	-0.350	-0.163	0.076	-0.040	0.054	0.041	-0.193	-0.053	0.041	-0.162	-0.025
	P	0.839	**0.015**	0.182	0.596	0.801	0.653	0.784	0.210	0.684	0.750	0.264	0.829
Sexual Abuse	β	-0.083	-0.100	-0.091	-0.148	-0.233	-0.168	0.275	-0.082	0.085	0.044	-0.172	-0.060
	P	0.559	0.499	0.360	0.302	0.129	0.083	0.061	0.592	0.414	0.735	0.231	0.515

^*^ Multiple linear regression (MLR) was used for correlational analysis to control age and BMI in the MDD and HC group, to control age, BMI, and diagnosis in all participants; plasma complements and CRP were LN-transformed in MLR

*C3* Complement component 3, *C3a* Complement component 3a, *C1q* Complement component 1q, *CRP* C-reactive protein, *MDD* Major depressive disorder, *HC* Health control, *ALL* All participants, *CTQ* Childhood trauma questionnaire

Spearman correlation analysis indicated that other clinical variables, including onset age of MDD, duration of illness, HAMD-24 score and HAMA score, were not associated with the levels of C3, C3a, C1q and CRP in the MDD group (Table S1), while after controlling for BMI and age levels of C3 was found to be associated with HAMA score (Table S1). Furthermore, correlations among the levels of plasma C3, C3a, C1q, and CRP were analyzed. Higher levels of plasma C3 and C3a were correlated with elevated CRP levels in the MDD group, but not in the HC group. However, peripheral levels of C3 were found to be positively correlated with C1q levels in the HC group (Table 6).

**Table 6:** Correlations among the levels of plasma C3, C3a, C1q and CRP in the MDD group and HC group

	MDD^#^				HC^#^			
	C3	C3a	C1q	CRP	C3	C3a	C1q	CRP
C3	1.000	─	─	─	1.000	─	─	─
C3a	0.131	1.000	─	─	0.210	1.000	─	─
C1q	0.204	-0.154	1.000	─	0.418*	0.186	1.000	─
CRP	0.321*	0.285*	0.091	1.000	0.238	0.069	0.194	1.000

*C3* Complement component 3, *C3a* Complement component 3a, *C1q* Complement component 1q, *CRP* C-reactive protein, *MDD* Major depressive disorder, *HC* Health control

^#^ Spearman correlation analysis was performed and spearman r is present in the table

^*^ *P* < 0.05

## Discussion

In this study, we found that peripheral level of C3 and its active product, C3a, in medication-free MDD was significantly higher than that in HC; whereas the concentration of plasma C1q and CRP in depressed patients was comparable to that in HC. Childhood trauma experiences were not associated with levels of C3, C3a, C1q and CRP in MDD patients, while C1q was associated with childhood emotional abuse and C3 was associated with childhood CTQ total scores, emotion abuse, emotional neglect, and physical abuse experiences in the HC group. Levels of C3 was found to be associated with HAMA score in MDD patients, while other clinical variables were not associated with complementary components and CRP. Additionally, levels of C3 and C3a were correlated with BMI in the MDD group, but not in the HC group. CRP was associated with BMI both in the MDD and the HC group.

Our data showed increased peripheral C3, and its active product, C3a levels in medication-free MDD compared to HC. C3 is a key component in the activation of complement cascade and is cleaved into two active molecules, C3a and C3b to exert its effect. Although peripheral C3 in MDD is frequently investigated in previous studies, to our knowledge, no previous studies have examined C3 and C3a at the same time. Our result provides further evidence for the existence of complement system activation in MDD patients and suggests that increased C3 may contribute to the pathophysiology of MDD through C3a. Supporting this view, in animal study C3 and C3a have been shown to significantly enhance the amount of infiltrating peripheral cells in the CNS and increase neuroinflammation [25, 38]. It should be noted that increased peripheral C3 in MDD has only been reported in some studies [14–17], but not in others [18–22]. The inconsistence may be related to the heterogeneity of MDD itself and the potential confounding variables which were not well-controlled in these studies. Taken together, these results suggest that increased peripheral C3 and C3a levels may be an important pathophysiological change in MDD, and C3 and C3a have potential to serve as biomarkers for MDD.

Our result indicated that the concentration of plasma C1q in depressed patients was comparable to that in healthy controls. Recently, two studies reported higher peripheral levels of complement C1q in patients with depression than that in the healthy controls with a small effect size [39, 40]. Although the reason for this inconsistence between the present study and the previous ones remains unknown, the differences on demographic and clinical variables between ours and previous studies should not be ignored, including age of patients and the severity of their depressive symptoms, which have been associated with the levels of complement components [20, 41]. It is well known that C1q is the initial component of the classic pathway of complement activation, thus our result indicates that the classic pathway may not be involved in the activation of complements in MDD. In contrary, it has been suggested that the alternative pathway may be dysregulated in MDD as the components in this pathway, like complement factor H, has been found altered in peripheral blood of MDD [17, 20, 42]. Further study should be carried out to determine whether classic or alternative pathway or both are related to pathophysiology of MDD.

Our data showed that the peripheral CRP was correlated with BMI in both MDD patients and HC. Several previous studies suggests that BMI may be a significant confounding factor mediating the association of some inflammatory factors, such as CRP and IL-6, with MDD [29, 43–45]. In line with these studies, we did find that the difference on the plasma levels of CRP between MDD and HC was disappeared after controlling BMI and age in the current study. These results indicate that BMI may play a more important role than MDD in explaining the peripheral levels of CRP. Thereof, future study examining the relationship between inflammatory factors and depression should consider the potential mediating effect of BMI.

Interestingly, our results exhibited that the peripheral levels of C3 and C3a both strongly correlated with BMI only in MDD group, but not in HC group. C3 and C3a were higher in the MDD group than that in the HC group even after controlling the potential confounding effects of BMI and age. These results suggest that unlike CRP, the association of complement C3 and C3a with MDD are not mediated by BMI. It is well known that BMI is an index of body fat mass. Adipose tissue has been recognized as an important endocrine organ and adipocytes, especially white adipocytes, are one of the main sources of circulating inflammatory factors including complement C3 [46]. We speculate that adipocyte dysfunction might contribute to the increasement of peripheral C3 and C3a in MDD. Supporting this view, the changes of the amount of intra-abdominal and pericardial adipose tissue [47–49] and adipocyte-derived factors like adiponectin and leptin [50, 51] have been observed in MDD patients. Further studies are required to replicate this result and explore the potential association between adipocytes dysfunction and complements in MDD.

Previous studies have shown that childhood trauma is associated with adult peripheral levels of inflammatory factors [30, 31]. However, the association between childhood trauma and adult complement components are seldom reported. Only one recent study reported that peripheral C1q levels were not associated with traumatic life events [40]. However, in that study the authors did not clearly define in which life stage the traumatic life events were experienced by MDD patients. Therefore, to our knowledge, our study is the first one to investigate the association between childhood trauma and complement components. Our data revealed that childhood traumatic experiences were not associated with adult peripheral levels of complement C3, C3a, C1q and CRP in MDD patients. In spite of this negative result, the relationship between childhood trauma and adult complement components is not definitive, as we found C3 and C1q were associated with childhood trauma experiences in the healthy controls. One plausible explanation for this discrepancy is that MDD patients usually present significant pathophysiological changes in multiple systems, such as nervous system, immune systems, and endocrine systems, and show dysregulated homeostasis status (weight changes, sleep disorders, and so on). All these pathophysiological and homeostatic abnormities may influence the peripheral levels of C3 and C1q, thus may mask the potential correlations of C3 and C1q with CTQ total and subscale scores as observed in the HC populations. Supporting this view, we found a significant correlation of peripheral levels of C3 with BMI in the MDD group. Additionally, the symptoms of MDD may also modulate the plasma levels of complement factors as the correlations between depressive symptoms with levels of complements have been found in some previous studies [40]. In the present study we also found a significant association of plasma C3 levels with HAMA scores. Furthermore, one animal study found that early-life stress caused C3 alteration in adulthood [52]. Childhood and adolescence are key stages for the development of central nervous system and immune system, which may be affected by stress depending on its timing, severity, and type. The long-term impact of childhood trauma on adult complement activation still requires more investigations in the future.

We did not find associations of peripheral levels of C3, C3a, C1q and CRP with the severity of depressive symptoms and other clinical variables such as the onset age and duration of disease in the MDD group. However, we did find an association of C3 levels with HAMA scores after controlling age and BMI. These results are consistent with some of previous studies where complements and CRP were found not to be correlated with the severity of depressive symptoms [53, 54], but inconsistent with others [40]. Our results also showed that levels of C3 and C3a positively associated with CRP levels in the MDD group, but not in the HC group, which suggests that patients with high concentrations of plasma C3 tend to present severe inflammatory state and anxious symptoms. However, our result can only be viewed as preliminary since the sample size is limited. Further investigation with large samples will be helpful to clarify this issue.

A major strength of our study is that all depressed subjects here are medication-free for at least two weeks, which to a large extent reduced the influence of pharmacotherapy on the evaluation of complements [55, 56]. The limitations in our study include, (1) the sample size is relatively small thus did not allow further analyses relevant to some of influencing factors; (2) childhood trauma experience is retrospectively self-rated and could be biased by recall errors; (3) other complement components such as C3b and C4, and components involved in alternative pathway such as complement factor H were not measured; (4) this is a cross-sectional study, which is unable to provide comprehensive information in a dynamic process; (5) the controls were hospital employees and college students, which may increase the systematic errors and limit the generalizability of results.

## Conclusion

In conclusion, our study showed significant higher levels of complement C3 and C3a in MDD, which suggest that complement C3 and C3a may play an important role in the pathophysiology of MDD. However, the effect may be modulated by many other biological and environmental factors in adult MDD patients in whom significant association between peripheral complement components, including C3, C3a and C1q, and stressful experience during childhood may be absent.

## Supplementary Information

**Additional file 1:** **Table S1.** Association of plasma complements and CRP with clinical variables in the MDD group.

## Data Availability

All data generated or analyzed during this study are included in this published article.

## Abbreviations

MDD: Major depressive disorder
CRP: C-reactive protein
IL-6: Interleukine-6
HC: Healthy control
HAMD: Hamilton rating scale for depression
HAMA: Hamilton rating scale for anxiety
CTQ: Childhood trauma questionnaire
ELISA: Enzyme linked immunosorbent assay
BMI: Body mass index
