# Supplementary material for: Plasma complement C3 and C3a are increased in major depressive disorder independent of childhood trauma
Source: BMC Psychiatry. 2022 Nov 29;22:741. doi: 10.1186/s12888-022-04410-3 (PMC9706857; doi:10.1186/s12888-022-04410-3)
Supplement: Supplementary file 1 — Additional file 1: Table S1. Association of plasma complements and CRP with clinical variables in the MDD group. [file 12888_2022_4410_MOESM1_ESM.docx]

Table S1. Association of plasma complements and CRP with clinical variables in the MDD group

|  | C3 | | C3a | | C1q | | CRP | |
| --- | --- | --- | --- | --- | --- | --- | --- | --- |
|  | r_s_/β | P | r_s_/β | P | r_s_/β | P | r_s_/β | P |
| Spearman correlation* |  |  |  |  |  |  |  |  |
| HAMD | 0.128 | 0.381 | -0.150 | 0.304 | 0.139 | 0.341 | -0.137 | 0.346 |
| HAMA | 0.107 | 0.339 | 0.040 | 0.787 | 0.139 | 0.339 | -0.076 | 0.606 |
| Onset age of MDD | 0.043 | 0.767 | 0.070 | 0.633 | 0.187 | 0.198 | 0.198 | 0.173 |
| Duration of illness | -0.261 | 0.070 | -0.017 | 0.906 | 0.043 | 0.771 | -0.157 | 0.280 |
| Multiple linear regression controlling age and BMI^#^ |  |  |  |  |  |  |  |  |
| HAMD | 0.212 | 0.131 | -0.060 | 0.679 | 0.092 | 0.538 | -0.095 | 0.468 |
| HAMA | 0.299 | **0.027** | -0.060 | 0.669 | 0.108 | 0.462 | -0.003 | 0.984 |
| Onset age of MDD | 0.479 | 0.156 | -0.184 | 0.594 | 0.311 | 0.385 | -0.149 | 0.638 |
| Duration of illness | -0.193 | 0.224 | 0.075 | 0.646 | -0.119 | 0.480 | 0.015 | 0.920 |

* Spearman correlational analysis was performed and spearman r (r_s_) is present in the table.

^#^ Multiple linear regression (MLR) was used for correlational analysis to control for age and BMI, and standardized beta (β) is present in the table; Plasma complements and CRP were LN-transformed in MLR. C3: complement component 3; C3a: complement component 3a; C1q: complement component 1q; CRP: C-reactive protein; MDD: major depressive disorder.
